# Evaluating factors that influenced the successful implementation of an evidence-based neonatal care intervention in Chinese hospitals using the PARIHS framework

**DOI:** 10.1186/s12913-022-07493-6

**Published:** 2022-01-25

**Authors:** Jieya Yue, Jun Liu, Yingxi Zhao, Sarah Williams, Bo Zhang, Lin Zhang, Qiannan Zhang, Xin Liu, Stephen Wall, Gengli Zhao

**Affiliations:** 1 Peking University First Hospital, 1 Xi’anmen St, Xicheng, Beijing, China; 2 Nuffield Department of Medicine, University of Oxford, Oxford, UK; 3 Save the Children UK, London, UK; 4 Save the Children, Chengdu, China; 5 Save the Children US, Washington DC, USA

**Keywords:** Evidence-based, China, Implementation, Context, Culture, Facilitation, PARIHS, Kangaroo mother care

## Abstract

**Background:**

Evidence based interventions (EBIs) can improve patient care and outcomes. Understanding the process for successfully introducing and implementing EBIs can inform effective roll-out and scale up. The Promoting Action on Research Implementation in Health Services (PARIHS) framework can be used to evaluate and guide the introduction and implementation of EBIs. In this study, we used kangaroo mother care (KMC) as an example of an evidence-based neonatal intervention recently introduced in selected Chinese hospitals, to identify the factors that influenced its successful implementation. We also explored the utility of the PARIHS framework in China and investigated how important each of its constructs (evidence, context and facilitation) and sub-elements were perceived to be to successful implementation of EBIs in a Chinese setting.

**Method:**

We conducted clinical observations and semi-structured interviews with 10 physicians and 18 nurses in five tertiary hospitals implementing KMC. Interview questions were organized around issues including knowledge and beliefs, resources, culture, implementation readiness and climate. We used directed content analysis to analyze the interview transcript, amending the PARIHS framework to incorporate emerging sub-themes. We also rated the constructs and sub-elements on a continuum from “low (weak)”, “moderate” or “high (strong)” highlighting the ones considered most influential for hospital level implementation by study participants.

**Results:**

Using KMC as an example, our finding suggest that clinical experience, culture, leadership, evaluation, and facilitation are highly influential elements for EBI implementation in China. External evidence had a moderate impact, especially in the initial awareness raising stages of implementation and resources were also considered to be of moderate importance, although this may change as implementation progresses. Patient experience was not seen as a driver for implementation at hospital level.

**Conclusion:**

Based on our findings examining KMC implementation as a case example, the PARIHS framework can be a useful tool for planning and evaluating EBI implementation in China. However, it’s sub-elements should be assessed and adapted to the implementation setting.

**Supplementary Information:**

The online version contains supplementary material available at 10.1186/s12913-022-07493-6.

## Introduction

The implementation of evidence-based interventions (EBIs) is usually a complex and multifaceted process. Bridging the evidence-practice gap enables improvement in the quality and effectiveness of health services and care [1, 2]. A range of facilitators and barriers that affect this gap have been identified in implementation research, these include organizational structures and processes, group dynamics and leadership, knowledge and beliefs as well as broad political, economic and socio-cultural factors [3]. There is increasing recognition and use of theories, models and frameworks in implementation science that draw on other disciplines such as psychology and sociology, to help bridge the evidence practice gap. These frameworks can be used to guide the implementation process, evaluate implementation outcomes, and support the systematic management of facilitators and barriers to reduce the evidence-practice gap [4]. Frameworks used for implementation usually include steps to identify implementation barriers and facilitators relevant to the context of practice. Most of these frameworks are based on individual or organizational change, climate, culture and leadership, examples include the Consolidated Framework for Implementation Research (CFIR) and Promoting Action on Research Implementation in Health Services (PARIHS) [5, 6].

The Promoting Action on Research Implementation in Health Services (PARIHS) framework was initially published in 1998 and is frequently used in implementation research [5]. The PARIHS framework conceptualizes the successful implementation of interventions using three interacting constructs, these are evidence, context and facilitation [7, 8]. Each construct has sub-elements: evidence includes research, clinical and patient experience; context includes culture, leadership and evaluation; and facilitation, the process of enabling implementation [9–11]. The framework requires the placement of each sub-element on a low to high continuum, an element placed highly on the continuum is considered conducive to successful implementation. The PARIHS framework can be used retrospectively to aid understanding of how the constructs and sub-elements of the framework have acted as barriers or enablers to successful implementation [12].

Since its initial publication in 1998, the PARIHS framework has been further tested, developed, and refined. In 2015 a revised version, the integrated or i-PARIHS framework was published, it defines successful implementation primarily as the achievement of implementation or project goals resulting from the facilitation of implementation with recipients, individuals or groups, acting in their context (local, organizational and health system). The core constructs of i-PARIHS are facilitation, innovation, recipients and context, facilitation is seen as an active process that responds to the three other constructs and enables implementation [13].

PARIHS has been used primarily in high-income countries, a recent citation analysis of 367 published papers utilizing the PARIHS framework found that approximately three quarters of these studies were conducted in the USA, UK, Canada and Sweden, very few took place in low- or middle-income countries and only one was conducted in China [14].

The process for successful introduction and implementation of EBIs in China differs from other countries. The values embedded in traditional Chinese culture continue to influence the ways in which people think and behave, which effects the way healthcare is organized and services delivered [3]. Due to the deeply rooted respect for social hierarchy, Chinese public hospitals usually have centralized organizational processes [15]. Chinese traditional values of harmony, benevolence, respect for authority and obedience to superiors can deter medical staff from changing their practice independently, especially if they perceive themselves to have little decision-making autonomy and low professional status.

To understand if and how the PARIHS framework could be used to guide successful implementation of EBIs in China we used the example of Kangaroo Mother Care (KMC), an EBI implemented in high-, low- and middle-income countries and recommended by WHO as an effective intervention to improve the survival and promote the growth and development of preterm newborns. KMC has only recently been introduced in China (Table 1). We retrospectively analyzed the contents of qualitative interviews conducted during the introductory stage, to investigate how the constructs and sub-elements of the PARIHS framework could be applied in China. Whilst the introduction of KMC in China was not guided by specific implementation models, it did take into account factors, included in many models, that influence the success of implementation including the evidence behind the intervention, the context and situation within the neonatal units and the training and support needed for the implementation of KMC. In our analysis we focused on the process of implementation roll-out rather than the intervention itself, which we have previously examined [16]. We used the original PARIHS framework instead of i-PARIHS as the former has clearly defined constructs, sub-elements, and rating criteria and better fits with our objective. In this study, we used KMC as an example of an evidence-based neonatal intervention recently introduced in selected Chinese hospitals, to identify the factors that influenced its successful implementation; we also explored the utility of the PARIHS framework in China and investigated how important each of its constructs (evidence, context and facilitation) and sub-elements were perceived to be to successful implementation of EBIs in a Chinese setting. Findings from this study could be used to inform strategies for introducing and implementing similar neonatal interventions such as family-centered care and nurturing care in Chinese hospitals.

**Table 1:** China’s premature birth intervention program and kangaroo mother care

Previously KMC was not practiced as part of routine neonatal care in China. Since 2014, the National Health Commission of China and China’s Premature Birth Intervention Program have been working to raise awareness and promote the implementation of KMC across a network of 50 hospitals. Ten of these hospitals volunteered to participate in a pilot of KMC implementation. Representatives from each hospital took part in a short theoretical and practical training in 2015. Prior to this, hospitals had either not implemented KMC or provided it only occasionally to individual newborns. Other activities to promote the implementation of KMC included international and national expert meetings, study/exposure visits for senior practitioners and policymakers to high income countries implementing KMC (UK, US, Netherlands and Sweden), KMC stakeholder workshops in China involving nurses, doctors and other cadres of medical staff and trainings provided to different cadres of health workers by Chinese neonatal care experts. Draft guidelines for KMC implementation were produced by a multi-stakeholder group. From 2017 to 2019, eight of the original ten pilot hospitals volunteered to use these standardized KMC guidelines to inform their further development and finalization. By the end of the pilot’s first year, nearly 20% of all preterm newborns born in the eight hospitals received intermittent KMC. Our analysis focuses on KMC role out at individual hospital level, not at national level.	

## Methods

### Study design and setting

This study is nested in a larger piece of mixed-method implementation research of KMC in China, qualitative data was gathered between August and September 2018, consisting of clinical observation performed once in each ward and semi-structured interviews. Data was gathered from five NICU wards and two postnatal wards located in five tertiary hospitals. Hospitals were representative of different geographic locations, economic development levels, and cultural backgrounds in China.

### Data collection

Data from clinical observations were collected by six research assistants (RA), once at the start of the site visit to each hospital using a standard observation form (see Additional file 1) including unit physical and human resource, neonatal care process related to breastfeeding and KMC. RAs asked clarifying questions to medical staff as necessary and appropriate to facilitate accurate documentation.

A semi-structured interview guide was developed, originally informed by the Consolidated Framework for Implementation Research (CFIR) [17], modified during training and pilot testing to suit the local setting and ensure that questions could be understood by participants. Interview questions were broad, open-ended and included targeted and prompting questions focused on issues including knowledge and beliefs, perception of the source of intervention, resources, culture, implementation readiness and climate (see Additional file 2). Using purposive sampling, RAs interviewed 18 nurses three of whom were facilitators, 10 doctors and 10 parents of newborns receiving KMC. Before conducting interviews, we invited the coordinators of each ward, usually the head nurse, to help select the interviewees, stipulating those interviewees be full-time nurses and doctors on site, and parents with experience of providing KMC. We interviewed two to three nurses, one to two doctors and one to two parents from each NICU and ward involved in the study. Where the head nurse was also the facilitator, we included them as an interviewee. Detailed characteristics of the interviewees are presented in Table 2. Interviews were conducted in private rooms using Mandarin, and each lasted 30-40 min. Regular meetings among the RAs were set up during data collection to monitor progress and discuss preliminary findings for quality assurance. Data saturation was reached at the end of our fifth hospital visit.

**Table 2:** Basic characteristics of the medical staff interviewed

ID	Hospital	Type	Dept	Education	Rank	Tenure	Training in KMC
1	A	Nurse	Obstetrical	Bachelor	Senior	14	Y
2	A	Nurse	Pediatric	Bachelor	Junior	4	Y
3	A	Doctor	Obstetrical	Master	Senior	16	N
4	A	Doctor	Pediatric	Master	Senior	28	N
7	A	Nurse	Obstetrical	Bachelor	Senior	14	Y
8	A	Nurse	Obstetrical	College	Junior	3	Y
9	A	Nurse	Pediatric	Bachelor	Junior	6	Y
10	A	Nurse	Pediatric	Bachelor	Junior	8	Y
11	B	Nurse	Pediatric	Master	Junior	8	Y
12	B	Nurse	Pediatric	Bachelor	Senior	N/A	Y
13	B	Doctor	Pediatric	Doctorate	Junior	5	Y
14	B	Doctor	Pediatric	Doctorate	Junior	3	Y
17	C	Nurse	Pediatric	Bachelor	Junior	9	Y
18	C	Nurse	Pediatric	Bachelor	Senior	10	Y
19	C	Doctor	Pediatric	Master	Junior	1	Y
20	C	Doctor	Pediatric	N/A	Senior	17	N
23	D	Nurse	Pediatric	Bachelor	Senior	9	Y
24	D	Nurse	Pediatric	Bachelor	Senior	12	Y
25	D	Doctor	Pediatric	Master	Junior	6	Y
26	D^a^	Nurse	Pediatric	Bachelor	Senior	28	Y
27	D	Nurse	Obstetrical	Bachelor	Senior	14	Y
28	D	Nurse	Obstetrical	N/A	Junior	4	Y
29	D	Doctor	Obstetrical	Bachelor	Junior	6	Y
30	D^a^	Nurse	Obstetrical	Bachelor	Senior	18	Y
33	E	Nurse	Pediatric	Bachelor	Junior	9	Y
34	E^a^	Nurse	Pediatric	N/A	Senior	23	Y
35	E	Doctor	Pediatric	Master	Senior	16	N
36	E	Doctor	Pediatric	Bachelor	Junior	7	Y

^a^Indicates facilitator role during facilitation

### Data analysis

As the main objective of this study is to use KMC as an example and explore the factors that influence the implementation of evidence-based interventions at hospital level utilizing the PARIHS framework, we focused our coding and analysis on interviews with nurses and physicians. We compiled information collected from clinical observations (ward contexts and KMC processes) and interviews with parents (their experience and attitude towards KMC) for use as contextual data. We chose to exclude information from parents in the analysis as this tended to focus on individual level barriers and facilitators and our primary interest for this analysis was factors related to hospital level implementation.

Semi-structured interviews were audiotaped and transcribed, with all personal identifiable information removed and replaced by unique anonymous codes. After two coders read through the transcripts and carried out some inductive coding, we found that PARIHS rather than CFIR best fit our data as the framework to explain the key factors for the successful implementation of KMC in China. We used directed content analysis to analyze the interview transcripts, which is commonly used to validate or conceptually extend a theoretical framework or theory. In our analysis we started with the original PARIHS framework to assess factors impacting implementation of KMC as an EBI. Although the PARIHS framework was revised to the i-PARIHS framework in 2015, we chose to use the original framework because the original version fitted our data better and there was a clear rating criteria. Two coders independently coded the transcripts using the constructs and sub-elements outlined in a paper by Rycroft-Malone [18]. New themes emerged from the coding analysis that did not fit in the original framework, we amended the original list of sub-elements through an iterative process the two coders individually analyzed the transcripts and then compared notes, a discussion was then held with all authors where additions to the sub elements were discussed and agreed. We highlighted the constructs and sub-elements that are considered most influential to implementation success in the study setting in order to inform optimal implementation strategies. The two coders independently rated the sub-elements on a continuum from “low (weak)”, “moderate” or “high (strong)” as outlined by Rycroft-Malone [9–11, 18]. For facilitation we rated the construct as a whole and the rating focused on “appropriateness” - for example “high” facilitation refers to its being appropriate to the needs of the particular change situation [11]. Ratings were compared and finalized after discussion between the coders to reach consensus. Criteria and supporting evidence for rating is presented in the Additional file 3. Preliminary findings were also presented to all other authors who were not directly involved in coding to triangulate and increase trustworthiness of the findings.

### Ethics approval

All methods were carried out in accordance with relevant guidelines and regulations. Ethical approval was obtained by Peking University First Hospital Biomedical Research Ethics Committee. Verbal informed consent was obtained from all interview participants.

## Results

All three constructs contributed to the successful implementation and roll-out of KMC in the five participating hospitals. The major themes that emerged for each construct and sub-element as well as their ratings are presented in Table 3. We recognize that some of the activities conducted as part of implementation roll-out could fit into more than one construct or sub element of the PARIHS framework. Where this is the case, we have selected the construct or sub-element we feel best fits the activity or the aspect of the activity which appears to be given more weight by the study participants.

**Table 3:** Major themes emerged and rating for PARIHS construct and sub-elements

Construct and Sub-element	Major theme emerged	Rating
**Evidence**		
**Research evidence**	- Local research ongoing, research focus on implementation not focusing on effectiveness	Low
**Clinical experience**	- Positive observation, feedback and small-scale data analysis from early practice	High
**Patient preference and experience**	- Some patient feedback, very limited patient preference	Low
**External evidence**	- Published literature to create early awareness but not fully applicable - Knowledge received from international, national and hospital-level expert training - Observed evidence from exchange visits from other countries (especially high-income countries)	Moderate
**Context**		
**Culture**	- Learning and communication culture through continuous training, communication between hospitals and among medical staff - Culture of multidisciplinary teamwork, between doctors and nurses, between obstetric and pediatric departments - Some opportunity for innovation	High
**Leadership**	- Strong support from leadership considered as pre-requisite, especially to tackle organizational resistance to change - Task-driven organizational structure led to fast resource mobilization and organizational changes	High
**Evaluation**	- Small scale data audit and feedback to maintain quality of implementation	High
**Resources**	- Limited physical environment and human resources constrained intervention scale-up - Financial resource and concern over out-of-pocket charges	Moderate
**Facilitation**		
**Purpose**	- “Task”: raising awareness, allocating resources, setting target and supporting staff	High
**Role**	- “Enabling”: moderate intervention initiation including disseminate training and allocate resource - “Practical”: supervision of practice	
**Skills and attribute**	- Varied attributes between facilitators, some more proactive in training and motivating staff	

### Evidence

Evidence as outlined in the PARIHS framework is derived from research, clinical experience, and patient preferences [9].

#### Research

We consider the “research” sub-element as locally generated research evidence from China. This is because whilst participants appreciated and understood the evidence for KMC from other countries, the context in which this evidence was generated was felt to be so different to the context in China that additional evidence from hospitals in China would be needed. This evidence was therefore considered under the sub element of “external evidence”. As implementation of KMC in China was very limited prior to the Premature Birth Intervention Program, such local research evidence was not available at the time of data capture. Therefore, research was not perceived as an important sub-element for implementation by study participants.

#### Clinical experience

After KMC was initiated in each hospital, clinical experience was considered important to continued roll-out. Direct experience and observation of clinical practice alongside results from small scale informal analysis of routinely collected data provided positive evidence. This evidence indicated that preterm infants who had received KMC had better health outcomes including breastfeeding, growth and development, stability of vital signs, and reduced hospital stay. One junior doctor stated:

*“At first, we were less confident and a little skeptical, because it’s a new intervention that we have not used before and we also did not know its benefit to the infants. Now we do this from Monday to Friday, and it indeed shorten hospital stay, this is for sure. The infants are feed better, KMC did make a difference.” [Junior doctor 25]*

Before the introduction of KMC medical staff had been worried about the effect it would have on incidence of nosocomial infections, however experience and small-scale data analysis showed no increase in infection rate. Additionally, medical staff stated that parents who practiced KMC had less anxiety and reported better bonding with their infants than parents who did not practice KMC. Medical staff reported improvements in their relationship with the parents of preterm newborns. Clinical experience provided medical staff with strong anecdotal evidence, and greater confidence in using KMC. Clinical experience was considered a very important sub-element for intervention roll-out.

#### Patient preference and experience

In our interviews with doctors and nurses, patient preference and experience did not appear to contribute to decision making on the introduction and further roll-out of KMC at hospital level. However, the doctors and nurses interviewed reported that most parents who were informed about KMC and asked if they wished to provide KMC to their premature newborns went on to provide KMC. Some nurses and doctors mentioned the patient experience as being positive and inspiring with one nurse stating:

*“You just tell them what the benefits are, not too deep as they might not understand, just simple words. When they know that it’s good for the infant, help maintaining their vital signs, they are quite happy that it can help the infant and promote breast milk secretion, they are quite happy and like to try KMC.” [Senior nurse 1]*

If parents had not been so willing to accept KMC it would have affected the results of the roll-out. However, there was limited mention of patient preference from study participants, and it was not perceived as a driver for hospital-level roll-out.

#### External evidence

External evidence was added as a new sub-element to the original framework. There were several kinds of external evidence considered of moderate importance for intervention initiation and implementation, including published literature, expert trainings, and study visits to other countries.

A small number of medical staff mentioned that they had obtained published research articles on KMC which sparked their interest in introducing KMC in their hospitals. After first hearing about KMC, some searched for further information online and created early awareness of the evidence supporting KMC. However, participants indicated that most published evidence came from countries with very different contexts to China and surmised that reported models of KMC were not applicable in their settings. Participants emphasized the need for guidelines adapted to China’s situation and the local hospitals’ setting. A nurse stated:

*“I searched some literature after I heard it first and know what it’s about. I think it’s in 2013 or so...But our experience mostly come from foreign countries, and during practice we somehow felt not very grounded.” [Junior nurse 11]*

Several participants mentioned that their knowledge of KMC came from trainings delivered by foreign experts or from study visits overseas. This training mainly referred to training attended by heads of wards or head nurses from each hospital that participated in centralized national level training delivered by experts from the UK and/or WHO. Training included the rational and evidence behind KMC, how to support mothers or other care givers to provide KMC, selection of newborns for KMC and care provision to the neonate during KMC. Nurses who participated in this training returned to their hospitals and cascaded training to nurses working in the NICU and postnatal wards. This cascaded training is included in the facilitation section.

Medical staff from a few hospitals were exposed to the evidence supporting KMC through exchange activities or communication with foreign experts in high-income countries (i.e., US and Germany) as part of long-term institutional relationships. This helped allay some of their fears about KMC implementation, for example medical staff were worried that the introduction of KMC may lead to an increase in nosocomial infection, rates, however by observing KMC in other countries, the hygiene measures taken and the safety of KMC implementation, medical staff gained confidence in this intervention and started to try out KMC for individual newborns in their own hospital achieving good outcomes. This type of exchange activity only occurred in a few of the participating hospitals. A senior doctor stated:

*“It was in 2016 when I went to the US and saw their implementation, previously I have never seen it, just heard of it. We went to [anonymous hospital] and everyone in the ICU could do it. After we communicated with them all our concerns and came back, the head nurse attempted to develop it in our unit. We also need constant communication with the foreign experts. We hold an annual neonatology international forum and foreign experts come to our hospital and visit. They give lectures on KMC as well, and we follow them gradually.” [Senior doctor 4]*

### Context

Context implies an understanding of the forces at work which give the physical environment a character and a feel. According to the original PARIHS framework, context has been subdivided into three core elements: an understanding of the prevailing culture, the nature of human relationships as summarized through leadership roles, and the organization’s approach to routine monitoring of systems and services i.e. evaluation [7, 10].

#### Culture

We identified several aspects of working culture considered crucial for implementation of EBIs that we rated “high” on the PARIHS framework. The culture of “learning” through continuous training and “communication” between hospitals and/or among medical staff were considered to positively impact the continuous roll-out of KMC. For example, hospitals communicated with each other on key challenges encountered during implementation, and some hospitals also established WeChat (a Chinese messaging app) groups to allow communication between different wards on improving the clinical practice of KMC. Interviewees stated:

*We communicate with other colleagues, and many provided assistance. I know [anonymous hospital] did very good, and I also visited [anonymous hospitals], they carried out KMC more. [Senior nurse 34]*

*We have a KMC group chat, whichever unit performed KMC, s/he will share how it was done, including gesture. But we are still trying. [Senior nurse 07]*

Participants also stated that the successful implementation of KMC was inseparable from the cooperation between “multidisciplinary teams”, including the cooperation between doctors and nurses and that between the neonatal ward and the postnatal ward. For each preterm infant, doctors and nurses often jointly decided on suitability for KMC. Such collaboration was especially important for late preterm infants on the postnatal wards where pediatricians had to evaluate the neonate before KMC practice could commence. Without multidisciplinary teamwork, it would have been very difficult to implement KMC.

#### Leadership

Study participants stated that the successful implementation of KMC could not be achieved without the support of hospital leaders. Implementation requires multi-faceted support including human resources, physical resources and policy change, the approval of senior management is considered a pre-requisite for tackling organizational resistance to change, and therefore intervention initiation and roll-out. The organizational structure and task-driven characteristics led to fast resource mobilization and change. The hospital leaders coordinated the allocation of resources and expedited the formulation of KMC policies and processes on the wards. Nurses and doctors emphasized the importance of leadership.

*“At first, we did not implement well, just one or two every month, not a large scale. The leaders emphasized this, and then it went well”. [Senior nurse 30]*

#### Evaluation

Formal routine data audit and supervision were reported to have effectively maintained the quality of KMC. Some hospitals designated staff for data collection, and the head nurse would monitor the implementation of KMC based on the data monthly leading to timely resolution of problems.

*“I think supervision is needed. Now we have a KMC group, and the lead nurse chose one that’s in charge of data reporting. Sometime the lead nurse will go and supervise in the middle of the month and see how KMC was done.” [Senior nurse 30]*

#### Resources

The availability of resources was frequently mentioned by participants as an important factor for implementation, it came out so strongly in our analysis that we believe it needs to be considered as an additional sub-element of the framework, within the construct of context.

The physical environment, primarily the lack of space and human resources were often cited as barriers for KMC implementation and scale-up. Where additional resources had been allocated e.g., recliners and privacy screens, roll-out of KMC accelerated. Limited space and materials were repeatedly cited as reasons for KMC not being provided to all eligible infants and for some hospitals limiting the time and/or frequency with which parents could provide KMC to their infants.

*“Our space is quite limited, and there’s not a large space. Another thing is that our staff number is limited, not enough nursing staff. There’s not much time and energy to take care of this”. [Junior nurse 10]*

Financial resources were a controversial factor and participants cited this as an area of concern for intervention sustainability. The implementation of KMC led to an increase in the use of hospital commodities (e.g., disinfection materials, protective gowns). While the further scale-up of KMC may be limited by financial expense, there was no consensus as to whether parents should be charged to cover the use of additional commodities, this meant that some hospitals charged, and some did not. Some participants expressed concern that charges might limit family members provision of KMC. Nurses spoke about the financial considerations of implementation.

*“We used to say that parents wanted to enter here. They have to wash their hands repetitively and would also ask you to change the diapers repetitively and then a lot of wet wipes and tissue papers are wasted…and also those isolation disposable gowns, we cannot charge for them. We understand that it’s good for the infants and could shorten hospital stay, but we feel like free labor.” [Junior nurse 10]*

*“Now for our unit we charge for KMC, but patients are very repelled by charges, when you mention this, they will think you only ask me to do KMC because you want to make money.” [Senior nurse 12]*

While resources were considered insufficient by study participants, it did not impede KMC’s initiation in hospitals. Medical staff were able to pilot KMC with existing or limited additional resources. The impact of lack of resources on sustainability and scale-up was the main concern from participants therefore we rated resources as a “moderate” sub-element.

### Facilitation

Facilitation is a technique by which one person makes things easier for others. The term describes the type of support required to help people change their attitudes, habits, skills, ways of thinking, and working. One of several change management strategies, it has received particular attention within nursing quality improvement and clinical practice development initiatives, and also in primary care audit [7, 11].

In the pilot of KMC implementation in China, department directors and head nurses from each hospital participated in theoretical and practical training, they were the first in their hospitals to receive KMC training. In addition, as heads of wards and departments, it was easier for them to mobilize resources and space, organize training, and draft hospital specific KMC policies to promote the implementation of KMC. They were not appointed as dedicated facilitators, even though they acted as facilitators, thus they were not funded and had no allocated facilitation time. We rated this construct as “high”, as facilitation was deemed to be appropriate to need, study participants emphasized the role of the head nurse in KMC implementation positively and described how the facilitation evolved as implementation progressed.

*“Our department has been working on KMC, and the hospital provided limited support. It’s always our head nurse and team lead who have been thinking of solutions.” [Senior nurse 18]*

#### Purpose

The purpose of facilitation in participating hospitals was positioned towards the “task” end of the task-holistic facilitation continuum. Facilitators raised awareness of KMC among medical staff, allocated available resources and tasks, set goals, and supported their achievement. Due to the task-driven nature of implementation, most hospitals set high targets for the number of newborns receiving KMC, and facilitators monitored progress to ensure targets were met.

#### Role

Study participants reported that the facilitators’ role changed as implementation progressed. In the initial stages of implementation facilitators focused on the “enabling” aspects of their role, including provision or oversight of training, planning of human resources and space, and drafting of hospital specific KMC policies and procedures. After the initial stages, facilitators played a more supervisory role in KMC practice and implementation. Facilitators also fostered peer support among nursing staff.

*“The head nurse has been considering these (physical location) questions, and how to standardize, how to appropriately charge… I also first heard of it (KMC) from the head nurse, and she provided training to us.” [Junior nurse 33]*

*“Now we allocate our job, one person in charge of eight patients, and there’s also one person in charge of coordination. If you cannot finish your work, you can ask for his support. We also have a peer support group, you help me, I help you, when you are busy, I will assist you. Now there’s more collaboration.” Senior nurse 07]*

#### Skills and attribute

There was much variation in the skills and attributes of different KMC facilitators and thus we could not define a clear set of skills essential to implementation facilitation within the hospitals. In one ward, a facilitator was proactive in visiting other hospitals to learn and adapt best practices locally. In another ward, a facilitator was able to identify a weakness in nurses’ skills and provided specific mentoring and training to build these skills.

*“I provided training the department… there’re some personal communication skills in it. When they talk about (KMC), they did not introduce in a step-by-step manner for parents as I suggested. You need to tell them the baby is preterm, we need to do what, and what’s the benefit. This is to inform you, if you are willing, we can provide this intervention.” [Senior nurse 07]*

## Discussion

We investigated the utility of the PARIHS framework in the context of neonatal and postnatal wards in five Chinese hospitals and summarized the factors that contributed to successful implementation of an EBI in China’s hospital setting. We used the introduction of KMC to these hospitals as an EBI example and analyzed qualitative data according to the PARIHS framework. The organizational culture and structure of Chinese hospitals differs from hospitals in other countries; therefore, the results of our study not only provide practical recommendations for the implementation of EBIs in China but add to the global literature on use of the PARIHS framework and how the process for successful EBI implementation could differ according to settings.

### Factors perceived to have a strong effect

Participants reported clinical experience to be an important source of evidence with a strong effect on EBI implementation in China. Despite robust global evidence of KMC’s benefits, medical staff were hesitant prior to implementation due to safety concerns and organizational resistance to change, partially because the studies had not taken place in China. Initial small-scale implementation of KMC built their confidence as they observed no negative side-effects and so expanded their practice. The experience of providing KMC services effectively reinforced implementation. This finding is similar to that of another study which found that nurses working in NICUs in China practiced developmental care based on their clinical experience rather than their educational experience [19].

Context including leadership, culture and evaluation are important factors in the implementation of EBIs. In China the context of implementation differs from Western countries specifically the sub-elements of leadership and culture. Chinese public hospitals use internal and centralized forms of control [15, 20], with hierarchical top-down management systems. Nurses work in a hierarchical system within a culture of obedience to clinical authorities, therefore successful implementation of EBIs is only possible when the managerial-level embraces evidence and takes action [3, 21]. Full support from hospital management such as head nurses makes it easier to allocate resources, provide training, define roles and responsibilities and foster communication and engagement among the entire clinical team [22–24], activities essential to implementing EBIs and emphasized by participants in our study.

Study participants considered the culture of multidisciplinary teamwork between obstetric and pediatric departments, and doctors and nurses to have had a strongly positive effect on KMC implementation. Close communication and collaboration between teams enabled timely reflection and sharing of problems and experiences during implementation, and made the implementation process more effective, similar findings were found in a study of cardiovascular care in Chinese hospitals [23]. This multidisciplinary approach was emphasized in other studies of neonatal EBI implementation including family centered care and developmental care [19, 25] the importance of multidisciplinary teamwork is not unique to China and can be found in other studies [26, 27].

Our study suggests that monitoring and evaluation play a key role in EBI implementation. Hospitals regularly collected data for formal audit (as opposed to informal analysis under “clinical experience”) and positive feedback from the audit acted as a driving force to promote further scale-up of KMC, and this is similar to the findings of others [28].

Participants reported that head nurses or their equivalents in each hospital assumed the role of EBI implementation facilitator they were deemed to be appropriate choices as facilitator. Their purpose was task-focused and included addressing operational issues such as training, resource management, policies formulation, data audit and evaluation, alongside “softer” issues e.g., creating and supporting peer learning and enabling multi-disciplinary teamwork. The combination and balance of different roles played by the facilitator to enable EBI has been reported in other settings and is listed in the PARIHS framework [11, 29]. In our setting, it was felt important to have a facilitator who is a hybrid clinical manager with good organizational knowledge, capable of exerting influence upwards, seeking senior leadership agreement, and promoting EBIs downwards to frontline staff, other studies had similar findings [30, 31]. Our findings suggest that identifying and supporting hybrid mid-level managers to facilitate EBI implementation has a strong positive effect on roll-out.

### Factors perceived to have a moderate effect

We categorized international expert training, published literature, international exchange visits as external evidence, because these were all based on information external to the Chinese context. Since KMC’s introduction is relatively recent in China there is a lack of evidence on KMC generated in China or in the Chinese language. Evidence from other countries was important for initial awareness raising about KMC. However, the intake of evidence rarely happens in its original form, and medical staff usually adapt the original guidelines or research findings to suit the particular situation, i.e. “tinkering” [13]. Participants suggested that experience from other countries cannot be fully applied to the situation within Chinese hospitals due to differing norms and ward setup, meaning that guidelines and protocols suitable for China need to be developed. Training from international experts seemed to be more conducive to the introduction of an intervention than other forms of external evidence. Medical staff preferred formal training to aid understanding of guidelines and documents and expressed interest in interacting with colleagues at higher levels during training. We believe external sources of evidence are most helpful when EBIs are new to a country and expertise cannot be found nationally, organizations should actively search for opportunities using external evidence to “expose” staff to EBIs in the initial phases of roll-out.

Many studies emphasis adequate resources as an important factor in intervention implementation [21, 22, 32]. While medical staff in our study mentioned less than desirable resources, we found that KMC could be implemented on a small scale through facilitation support with existing or limited additional resources, however resource limitations could be a barrier for further scale-up. According to the PARIHS framework, the relationship between available resources and implementation of EBIs is not straightforward, and resources need to be appropriately allocated and managed in order to influence the implementation process positively. Additionally, the focus on resources should not be at the expense of relationships, culture, and ways of working as all are needed for a holistic approach to implementation [24].

### Factors perceived to have a weak effect

Evidence from national research was not mentioned as having a strong effect on implementation, however this could be because at the time of data collection national research was in progress and results were not yet available, to compensate for the lack of national research medical staff used informal small-scale data analysis to gain evidence about KMC.

Participants reported that they had received positive feedback from parents since the implementation of KMC and that patient preferences influence implementation on an individual basis, however patient preference was not considered an important factor in decision-making for ward/unit-level implementation. The reasons for this are unclear, it could be due to the prevailing culture in China with patients generally hesitant to participate in decision-making or power sharing [33] and doctors often unaware of their patients perspectives [34]. More research is needed in China to understand patient preference and experience, clinician’s perception of patient preference and how both these factors could contribute to better implementation of EBIs.

### Methodological considerations

The PARIHS framework has been widely used as an organizing or conceptual framework to help explain and predict why the implementation of EBIs is or is not successful. There has been criticism of the framework including the lack of evidence from prospective implementation studies on its effectiveness, its focus on the facilitation role rather than the facilitation process and a lack of detail around its theoretical foundations [8]. A revised-PARIHS framework was developed with more constructs and over 30 characteristics [13]. In our study, we used the original PARIHS framework as it has been widely used and has clearly defined constructs, sub-elements, and rating criteria (the i-PARIHS framework lacks a rating criteria) and better fits with our objective of identifying the factors with a strong effect on EBI implementation. We used PARIHS as a guiding theory and added open coding during data analysis to capture emerging themes that did not fit the original framework. For example, we included “external evidence” and “resources” as two additional sub-elements under the constructs of evidence and context, respectively. The construct of evidence in the original PARIHS framework emphasized local research evidence alongside, patient experience. However, in this study we found that published literature and exchange visits to hospitals in other countries promoted an early awareness of and confidence in KMC when local research evidence was unavailable. This is similar to the sub-element of innovation from the revised i-PARIHS framework. Harvey and Kitson emphasize that *“people rarely take evidence in the original form of a systematic review or clinical guideline and directly apply it within an implementation project rather they incorporate evidence in a number of different ways, which typically involves adapting the original evidence in some way to suit their particular situation”* [11] - the original evidence is blended with practice-based knowledge and experience. Similarly, the original PARIHS framework did not emphasize the importance of resources, but in our analysis, we found that sufficient physical and financial resources are important for KMC to be further scaled up, therefore we added resources as a sub-element in the construct of context. This is in line with the inclusion of resources in the i-PARIHS framework within the construct of recipient [13]. We believe this approach addresses some of the critiques to the PARIHS framework and can better highlight the most important influencing factors in the Chinese context.

Regarding the utility of the original PARIHS framework in the Chinese context, we found that the proposed rating criteria was not always applicable to our setting. For example, the original rating for “culture” rated “task-driven organization” as weak/low. Considering the organizational culture in China we do not believe a task-oriented approach is detrimental to the implementation of EBIs in China. We recommend that researchers adapt the rating criteria to suit their situation when attempting to rate the sub-elements.

Several limitations should be considered when interpreting our results. We used KMC as an example of an EBI with which to test the utility of PARIHS to China, yet KMC is a unique intervention with its own characteristics that may affect the success of implementation. NICUs and postnatal wards also have differences when compared to other wards. Therefore, whilst our findings are useful for others intending to introduce and roll-out EBIs in China, the generalizability of our findings especially to other areas of care needs to be considered. Another limitation is that the number of staff per ward who took part in the interviews was relatively low and only formed a small proportion of the total number of staff per ward. So whilst we endeavored to select participants from different cadres with experience of KMC implementation our purposive sampling could have led to bias whereby selected participants could hold differing views about KMC implementation than their colleagues. Furthermore, KMC was only piloted in eight tertiary hospitals as part of the Premature Birth Intervention Program. While the five selected hospitals were representative of different geographic locations, economic development levels and cultural backgrounds, we were unable to further examine how these characteristics impacted KMC implementation. Additionally, we conducted our interviews in late 2018, 7 to 8 months into the formal pilot of KMC implementation, though the pre-implementation stages (creating awareness, committing to implement and preparing to implement) [35] had started in 2014. While we had evidence at the time of interview that approximately 20% of preterms were receiving KMC each month in all participating hospitals (unpublished data), we did not have evidence of KMC being fully integrated into routine practice or of its sustainability for example, hospital records suggest that provision of KMC stopped during the COVID-19 outbreak as an infection prevention measure and has now been resumed. We assessed the utility of PAHRIS at a specific stage of implementation and important elements of EBI implementation relevant to sustainability may not be captured in our analysis.

### Recommendations

Our results can be used to inform medical staff, program managers and policy makers planning the introduction and implementation of EBIs in Chinese hospitals. Our findings may also be of interest to policymakers in countries with a similar socio-cultural background especially those in other Asian countries. We recommend the following strategies be utilized when implementing EBIs:

1. Identify and support hybrid mid-level managers (head nurses, team leads, senior clinicians or nurses) to facilitate EBI implementation.
2. Secure senior leadership and organizational authorities’ commitment and support for EBI implementation early in the implementation process.
3. Use external evidence such as trainings provided by international experts, published literature and exchange visits to raise awareness and expose medical staff to existing evidence and practice when expertise in the intervention does not exist in country.
4. Contextually adapt international protocols and/or guidelines at national and local level to suit the implementation setting.
5. Promote effective multi-disciplinary team collaboration and communication, encouraging clinical experience sharing.
6. Conduct continuous monitoring, evaluation, supervision, and feedback on EBI implementation on a regular basis.

## Conclusion

Based on examining KMC implementation as a case example, the PARIHS framework is a useful tool for planning and evaluating EBI implementation. However, it’s sub-elements should be assessed and adapted to the implementation setting. Using the PARIHS framework as a guide for analysis we found clinical experience, culture, leadership, evaluation, and facilitation to be key elements for EBI implementation in China. External evidence had a moderate impact, especially in the initial awareness raising stages of implementation. Resources were also considered to be of moderate importance, although our analysis indicates that the importance of resources may increase as implementation progresses and the intervention is scaled-up. Our findings about the importance of different elements of the PARIHS framework can be used to inform the future roll-out and implementation of EBIs in similar clinical settings within China.

## Supplementary Information

**Additional file 1.** Kangaroo mother care qualitative study: clinical observation information.

**Additional file 2.** KMC interview guide for nurses and physicians.

**Additional file 3.** Rating criteria and rationale for PARIHS constructs and sub-elements.

## Data Availability

The datasets used and/or analyzed during the current study are available from the corresponding author on reasonable request.

## Abbreviations

EBI: Evidence-based intervention
KMC: Kangaroo mother care
NICU: Neonatal intensive care unit
PARIHS: Promoting Action on Research Implementation in Health Services
WHO: World Health Organization
