# Supplementary material for: Evaluating factors that influenced the successful implementation of an evidence-based neonatal care intervention in Chinese hospitals using the PARIHS framework
Source: BMC Health Serv Res. 2022 Jan 25;22:104. doi: 10.1186/s12913-022-07493-6 (PMC8787972; doi:10.1186/s12913-022-07493-6)
Supplement: Supplementary file 1 — Additional file 1. Kangaroo mother care qualitative study: clinical observation information. [file 12913_2022_7493_MOESM1_ESM.docx]

**Additional file 1. Kangaroo mother care qualitative study: clinical observation information**

|  | A - NICU | A - PNW | B - NICU | C – NICU | D - NICU | D - PNW | E - NICU |
| --- | --- | --- | --- | --- | --- | --- | --- |
| Number of rooms | 9 | 20 | 3 | 1 | 4 | 27 | N/A |
| Number of beds | 120 | 50 | 60 | 60 | 58 | 27 | N/A |
| Number of patients during observation | 84 | 55 | 72 | 91 | 55 | 27 | N/A |
| Number of preterm infants during observation (for PNW) | N/A | 1 | 47 | N/A | N/A | 1 | N/A |
| Number of doctors during observation | 10 | 12 | 30 | 20 | 10 | 2 | N/A |
| Number of nurses during observation | 32 | 15 | 19 | 18 | 19 | 10 | N/A |
| Doctor/bed ratio during observation | 1:12 | 1:4.2 | 1:2 | 1:3 | 1:5.8 | 1:13.5 | N/A |
| Nurse/bed ratio during observation | 1:3.8 | 1:3.3 | 1:3.2 | 1:3.3 | 1:3.1 | 1:2.7 | N/A |
| KMC implementation period | Separate KMC room open all day, NICU after 5 pm | All day | Morning and afternoon | Morning | Afternoon | Morning and afternoon | Afternoon |
| Family visit | Corridor | In-room | Camera before KMC, in-room after KMC | Camera | Camera | In-room | N/A |
| Quarantine measure required for family members when entering NICU | Shoe cover | N/A | Gown and shoe cover | Mask, shoe cover | Hat, mask and shoe cover, no phone allowed | N/A | Hat, mask, gown, shoe cover |
| KMC chairs | Regular bed | Regular bed | Reclining chair | Reclining chair | Reclining chair | Regular bed | Reclining chair |
| KMC gown | Parents’ own clothing | Hospital clothing | Parents’ own clothing | Parents’ own clothing | Parents’ own clothing | Parents’ own clothing | Parents’ own clothing |
| Number of KMC conducted during observation | 3 | 1 | 4 | 6 | 1 | 1 | 1 |
| How medical staff assist with KMC during observation | Posture adjustment, KMC benefit introduction, etc. | Oral instruction | Posture adjustment, KMC benefit introduction, temperature taking, etc. | Oral instruction, weight, feeding instruction | Not observed | Not observed | Oral instruction |
| Whether KMC and breastfeeding is recommended prior to discharge during observation | Not observed | Not observed | Yes | Not observed | Not observed | Not observed | Yes |

Note: Data was missing for hospital E NICU for the hospital characteristics.
