# Supplementary material for: Evaluating factors that influenced the successful implementation of an evidence-based neonatal care intervention in Chinese hospitals using the PARIHS framework
Source: BMC Health Serv Res. 2022 Jan 25;22:104. doi: 10.1186/s12913-022-07493-6 (PMC8787972; doi:10.1186/s12913-022-07493-6)
Supplement: Supplementary file 2 — Additional file 2. KMC interview guide for nurses and physicians. [file 12913_2022_7493_MOESM2_ESM.docx]

**Additional file 2. KMC interview guide for nurses and physicians**

**Personal information:** Age, sex, educational attainment, profession, title, years of working, KMC trainings received.

| Questions | CFIR Constructs |
| --- | --- |
| KMC Basic Information |  |
| - Do you know what’s KMC? - How does KMC influence premature infants’ growth? How does it influence parents? - What’s the procedure of KMC? | Knowledge and Beliefs About the Intervention |
| - Do you think KMC is complicated (duration, difficulty, procedure, charge)? | Complexity |
| - Who will you ask if you encounter any question? | Intervention Source |
| - Is KMC suitable for your daily work now? - How to integrate KMC into your daily work? - Does KMC promote your daily work? | Adaptability |
| - Is the workload of KMC one of your job performance criteria? - Has the lead doctor/nurse considered giving awards and recognition for KMC? | External Policy and Incentives |
| Examples of KMC |  |
| - Has anyone performed KMC today? - If yes, who made the decision to perform KMC? Why the infant was selected for KMC? Is there any other infant with similar condition that did not perform KMC? Why? - If no, why there’s no KMC performed today? When’s the last time KMC performed? | Intervention Source |
| KMC Training |  |
| - When and how did you first know of KMC? - How do the promotion materials influence you? - How was the training session? The pros and cons? Do you need more training? | Intervention Source  Knowledge and Beliefs About the Intervention |
| - At what stage are you aware of KMC intervention and utilization? | Individual Stage of Change |
| Resources Needed for KMC |  |
| - What basic infrastructure (design, equipment, personnel) will influence KMC implementation? | Structural Characteristics |
| - What do you think of the promotion materials (quality, clothing, mirror, cups, WeChat groups)? | Design Quality and Packaging |
| - What other resources are needed? | Patient Needs and Resources  Cost |
| - Do you think the current resource is adequate for KMC? | Patient Needs and Resources |
| - How to acquire those resources? What’s the challenge? - If inadequate, what other resource is needed from the hospital? Have you received any of those resource from hospital? What resource is easier to get? | Patient Needs and Resources |
| Acceptance among Hospital Colleagues and General Environment |  |
| - How do the hospital and ward leadership think of KMC? - What’s the goal for KMC implementation at the moment? How to achieve? - What’s the progress this month? Anyone in charge of data collection? - What’s the acceptance rate of KMC in the hospital and ward? Why? - Do you think your colleagues can assist parents to perform KMC? | Readiness for Implementation  Implementation Climate |
| - Does KMC provide comparative advantage for your hospital? | Peer Pressure |
| - What external policy could sustain KMC implementation? | External Policy and Incentives |
| - What’s the process of attitude change towards KMC? How did that happen? | Culture |
| Parents’ Feedback of KMC |  |
| - How many parents ask for KMC? How do they know of KMC? Wechat groups? - Why parents refuse KMC? Charge, location, emotion? - What’s their attitude towards KMC? - What’s parents’ understanding of KMC? - What’s parents’ feedback of KMC? Does that change doctor-patient relationship? | Patient Needs and Resources |
| - Do you have confidence for assisting parents to perform KMC? Why? | Self-Efficacy |
| Suggestions and Need for KMC |  |
| - During implementation, what changes are needed for KMC? - Do you think you can make those changes? Why and why not? | Patient Needs and Resources |
| - How to sustain the promotion of KMC in the wards? | Implementation Climate |
| - Do you think KMC should be scaled up? When should KMC be introduced to parents? Before or after delivery? | Implementation Climate |
| - Can you tell a story of collaboration with other departments/units during KMC implementation? | Networks and Communications |

Thank you for your participation!
