# Supplementary material for: Evaluating factors that influenced the successful implementation of an evidence-based neonatal care intervention in Chinese hospitals using the PARIHS framework
Source: BMC Health Serv Res. 2022 Jan 25;22:104. doi: 10.1186/s12913-022-07493-6 (PMC8787972; doi:10.1186/s12913-022-07493-6)
Supplement: Supplementary file 3 — Additional file 3. Rating criteria and rationale for PARIHS constructs and sub-elements. [file 12913_2022_7493_MOESM3_ESM.docx]

**Additional file 3. Rating criteria and rationale for PARIHS constructs and sub-elements**

| Construct and Sub-element | Rating criteria | Rating and rationale |
| --- | --- | --- |
| *Evidence* | | |
| *Research evidence* | - High: Perceived as strong, supported by well-conceived, designed and executed research - Medium: Perceived as descriptive or anecdotal - Low: Perceived as weak, not valued as evidence | **Low**   - Local research ongoing, research focus on implementation not focusing on outcome or effectiveness, not yet formalized as evidence |
| *Clinical experience* | - High: Mainly supportive, consensus within similar groups and value as evidence - Medium: Divided or support is mixed - Low: Mainly unsupportive, lack of consensus within similar groups | **High**   - Evidence generated from clinical practice and routine data analysis critically reflected upon |
| *Patient preference and experience* | - High: Incorporated in most aspects, partnership with patients - Medium: Incorporated in some aspects - Low: Not incorporated into any aspects, patients not involved, not valued as evidence | **Low**   - Patient narratives and experiences are seen as a moderate source of evidence, patient preferences not used as part of the decision- making process for unit level implementation (but useful for individual-level implementation) |
| *External evidence* | - High: Perceived as strong, experience reflected upon and seen as part of the decision - Medium: Perceived as descriptive or anecdotal - Low: Perceived as weak, not valued as evidence | **Medium**   - Research evidence from other countries were considered relevant less applicable - Experience from expert training and visit to high-income countries affirmed health workers of the intervention’s safety, prompted decision-making for implementation |
| *Context* | | |
| *Culture* | - High: Promotes learning organization, values individual and clients, local consensus is supportive, multiple opportunities for innovation - Medium: Local support is mixed, some opportunity for innovation - Low: Low regard for individuals, local consensus is unsupportive, limited opportunity for innovation. | **High**   - Despite the original framework by Jo Rycroft-Malone listed task-driven organization as “low”, we believe this is due to cultural difference - Cultures that pay attention to individual need, group processes and organizational structure through enabling learning, peer-support, communication, teamwork; some opportunity for innovation. |
| *Leadership* | - High: Strong organization, appropriate resource distribution, and well-defined roles - Medium: Mixed aspects of organization, resource distribution, and role definition. - Low: Poor organization, poor resource distribution, and undefined roles | **High**   - Effective organizational structure (in the Chinese setting) that enabled quick resource distribution and addressed organizational resistance to change |
| *Evaluation* | - High: Ongoing auditing of and/or feedback on group and/or individual performance. - Medium: Some auditing of and/or feedback on group and/or individual performance. - Low: No auditing of or feedback on group and/or individual performance. | **High**   - Ongoing routine data collection and analysis, supervision visits that provided feedback to medical staff, which maintained quality of implementation |
| *Resources* | - High: Appropriate resource that respond to need and supportive to implementation - Medium: Some resource but not sufficient - Low: Lack of resource and impeded implementation | **Medium**   - Physical and human resources appropriately allocated though not sufficient and impeded further scale-up. |
| *Facilitation* | | |
| *Purpose* | - High: Appropriate mechanism for facilitation - Medium: Some mechanism for facilitation - Low: No mechanism or inappropriate methods of facilitation | **High**   - Appropriate facilitation mechanism for the local setting, more on “task” spectrum that is focused on the achievement of tasks and goals, i.e. the “Oxford model”, less on the “holistic” spectrum - Less well-defined role, skills and attributes |
| *Role* |  |  |
| *Skills and attribute* |  |  |
